# 

**Published:** 1916-06-17

**Authors:** 


### CONTENTS.

| | PAGE |
|---|---|
| **The Military Service Acts and the Profession** | 247 |
| **The Certificate of the Cause of Death...** ... | 248 |
| **Hospital and Institutional News ...** ... ... | 249 |
| Our Hospital Sunday Number—Twenty-five Years as Hospital Chairman—The South London Hospital for Women—An Ulster Hospital and Incorporation—Charity on the South Coast—Federated Malay States Hospital—Nursing Tuberculosis in Hertfordshire—Further Gifts to Cardiff Hospital—Institutional Endowments Occasioned Through the War—King's College Hospital—Extension of Queen Margaret College—The Cavendish Lecture—The B.M.A. and the Remuneration of Poor-Law Medical Officers—In Place of the Annual Dinner—Is a Separate Chapel Essential for Worship?—The Compulsory Removal of Phthisical Patients—Coal-Dust and Phthisis—A Holiday Home as a School—The Reform of the Funeral—Scotland and Mentally Incapacitated Soldiers—Hospital Investments in War Loan—"*Blighty*: The Free Paper"—The May *Gazettes* from Cambridge—The R.A.M.C. Depôt Magazine—Coroners' Juries in War-time—The New Secretary of the Royal Hampshire Hospital—The War Standard of Sanitation—A Contumacious Council—This Week's Drug Market. | |
| **Internal Secretions and their Functions** ... | 255 |
| A New Chapter in Modern Physiology. | |
| **School Medical Inspection in the Transvaal...** | 256 |
| A Record of Progress. | |
| **The Institutional Treatment of Consumption** | 257 |
| IV.—Sanatorium Buildings and Equipment. | |
| **A Legal Quibble** ... ... ... ... ... | 258 |
| **A Medical Causerie** ... ... ... ... ... | 259 |
| **The Treatment of Wounded Officers** ... ... | 260 |
| The Royal Free Hospital and the War. | |
| **National Insurance Act Developments...** ... | 261 |
| The Interim Report of the Committee of Inquiry. | |
| **The Matrons' and Sisters' Department** ... | 263 |
| The General Nursing Council and College of Nursing: A Draft Registration Bill Agreed to. | |
| Round the Hospitals. | |
| British Ambulance Unit in Italy—A Ramsgate Memorial—Completing an Operation Theatre Unit—The Hospital Store-Room—The Pension Fund Report. | |
| **The Registration of Nurses** ... ... ... | 265 |
| First Draft of Agreed Bill. | |
| **Hospital Results in 1915** ... ... ... ... | 266 |
| **Passing Events and Topics...** ... ... ... | 266 |
| **The Editor's Letter-Box** ... ... ... ... | 267 |
| The English of Medical Writers—Economy in the Annual Report—The Feeding of Patients and Staff—The National Egg Collection for the Wounded. | |
| **Matrons' and Sisters' Appointments** ... ... | 268 |
| **Institutional Needs** ... ... ... ... ... | 268 |
| **The Institutional Worker** ... ... (Suppl.) | 1 |

